# Comparison of laparoscopic and open ileocecal resection for Crohn’s disease in children

**DOI:** 10.1007/s00383-023-05419-9

**Published:** 2023-02-27

**Authors:** V. Dotlacil, T. Lerchova, S. Coufal, B. Kucerova, J. Schwarz, O. Hradsky, R. Skaba, M. Rygl

**Affiliations:** 1 Department of Paediatric Surgery, Second Faculty of Medicine, Charles University and Motol University Hospital, V Uvalu 84, Praha 5, 150 06 Prague, Czech Republic; 2 Laboratory of Cellular and Molecular Immunology, Institute of Microbiology, Academy of Sciences of the Czech Republic, Prague, Czech Republic; 3 Department of Paediatrics, Second Faculty of Medicine, Charles University and Motol University Hospital, Prague, Czech Republic; 4 Department of Paediatrics, Faculty of Medicine in Pilsen, Faculty Hospital, Charles University in Prague, Pilsen, Czech Republic

**Keywords:** Paediatric surgery, Crohn’s disease, Ileocecal resection, Laparoscopy, Single-incision laparoscopic surgery

## Abstract

**Purpose:**

Ileocecal resection (ICR) is the most frequently performed surgery in paediatric Crohn’s disease (CD) patients. The aim of the study was to compare laparoscopic-assisted and open ICR.

**Methods:**

Retrospective review of consecutive CD patients undergoing ICR between March 2014 and December 2021 was performed. The patients were divided into open (OG) and laparoscopic (LG) groups. Compared parameters included patients’ demographics, clinical characteristics, surgery, duration of hospitalisation and follow-up. Complications were classified according to the Clavien–Dindo classification (CDc). Risk factors were identified using multivariable analysis.

**Results:**

Sixty-two patients (29 females, 46.7%) were included in the analysis, forty-two patients in OG. The median duration of surgery was 130 in OG versus 148 in LG (*p* = 0.065) minutes. Postoperative complications were reported in 4 patients (12.1%). There was no significant difference in postoperative complications according to CDc (OG 7.14 vs LG 5%, *p* = 1). The median length of hospitalisation was 8 in OG and 7 days in LG (*p* = 0.0005). The median length of follow-up was 21.5 months.

**Conclusion:**

The laparoscopic-assisted approach had shorter hospital stay and was not associated with increased risk of 30-day postoperative complications. Laparoscopic surgery should be considered the preferred surgical approach for primary ICR.

## Purpose

Crohn’s disease (CD) is a chronic, relapsing and remitting inflammatory condition that occurs in adults and children. Its incidence in the paediatric population is increasing worldwide. The phenotype is often characterised by extensive inflammation and an aggressive and progressive disease course [1–3].

Despite adequate conservative treatment, one-third of paediatric patients develop CD-related complications, such as fistula, stricture and obstruction, and require surgery within 5 years of their initial diagnosis [4]*.* Surgery may intervene the treatment process at any stage of the disease, and with a proper indication, timing and preoperative optimization, can induce immediate remission in patients. The most common intra-abdominal surgery is ileocecal resection [5]. According to the recent ECCO guidelines for adult patients [6], laparoscopic surgery should be offered as the first-line surgical approach for CD, dependent on appropriate expertise. Data demonstrating the superiority of the laparoscopic approach over the open approach in the paediatric population are still scarce; therefore, this kind of recommendation could not still be established for paediatric CD [7].

A cohort of paediatric CD patients treated at the authors’ institution was retrospectively analysed to compare results of the laparoscopic and open operative approach.

Given the data from the adult population [6], we assume that even within the child population, laparoscopic approach is associated with fewer postoperative complications.

## Methods

### Patient population

Medical records were reviewed to identify all paediatric CD patients (≤ 19 years of age) who underwent surgery at a single tertiary centre between March 2014 and December 2021. All patients were diagnosed with CD based on Porto criteria or revised Porto criteria [8, 9] and were treated according to currently valid European Crohn’s and Colitis Organisation/European Society for Paediatric Gastroenterology, Hepatology and Nutrition (ESPGHAN) guidelines [10, 11]. Patients who underwent surgery other than ileocecal resection or intestinal continuity was not restored after resection were excluded from the study.

### Surgical technique

Open surgery was performed via vertical midline laparotomy with ileocolic mobilisation and subsequent anteposition of the mobilised bowel segment through laparotomy. The next steps are the same for both OG and LG. Laparoscopy was initially done by three-port access (one umbilical, one left epigastric and one in the left iliac fossa, all 5 mm) and subsequently single incision laparoscopic surgery with “self-made” port (Alexis^®^, Applied Medical, Rancho Santa Margarita, CA and rubber glove) via vertical incision in umbilicus was performed. Laparoscopy started with assessment of the small and large bowel. The right colon, hepatic flexure and ileocecal region were mobilised laparoscopically with monopolar electrocautery, and the diseased segment was exteriorized through 2–3 cm extension of the umbilical trocar site. The mesentery was divided extracorporeally, the diseased segment was excised, and a primary ileocolic anastomosis was performed. After gaining sufficient experience with laparoscopy, a laparoscopic programme was launched in IBD surgery and since 2018, we have started surgery laparoscopically for all patients, and only in the event of an unfavourable intra-abdominal findings is there a conversion to open surgery.

### Study design

A retrospective analysis of medical charts was performed to evaluate association of therapeutic approaches (open and laparoscopic-assisted) with outcomes.

### Clinical variables of interest

The data set included age, gender, body mass index and medical history (corticosteroids or immunomodulators—azathioprine, mercaptopurine, methotrexate, and biologics). Disease duration, character, and history (disease behaviour according to the Paris classification), indication for surgery (acute complications that required emergency surgery—gastrointestinal bleeding, intestinal perforation or obstruction), timing of the operation, type of procedure performed, length of hospital stay, 30-day postoperative complications, and follow-up were analysed. Complications were identified during the hospital stay and/or within the postoperative follow-up.

### Outcomes

The primary outcome was 30-day postoperative complications with respect to operative approach. Complications were graded using the Clavien–Dindo classification (CDc) [12]. They were characterised specifically as surgical-site infections (SSIs; superficial, deep and organ/space), intra-abdominal septic complications (anastomotic leakage, intra-abdominal abscess or enterocutaneous fistula), non-SSIs, ileus (bowel obstruction with radiographic confirmation and requirement of a nasogastric tube for decompression or operative intervention), readmission and reoperation (ROR).

Secondary outcomes were length of hospital stay, postoperative pain, tolerance of peroral intake, duration of urinary catheter use, epidural analgesia, antibiotics, parenteral nutrition and central venous catheter.

### Statistical analysis

The continuous variables were tested for normality using the D’Agostino–Pearson normality test. The differences between laparoscopic-assisted and open groups were analysed with the Mann–Whitney test (continuous variables) or by Fisher’s exact test (dichotomous variables). Continuous variables are presented as median (range or interquartile range—IQR), and dichotomous variables as the number of cases (percentages), and *p* values of < 0.05 were considered statistically significant. GraphPad Prism statistical software (version 8.1.1, GraphPad Software, San Diego, CA, USA was used for statistical analyses. To account for potential confounding effects, we performed multivariate logistic regression analyses in the R statistics platform (R foundation for Statistical Computing, http://www.r-project.org).

### Ethical commission

This study was approved by the authors’ institutional ethical committee (EK-872/22).

## Results

In total, sixty-two patients (29 females, 46.7%) were included in the analysis, forty-two patients in OG. The median age was 17 years (IQR 14.8–17.4) at the time of operation and 14 years (IQR 11.1–15.9) at the time of diagnosis. Penetrating disease behaviour was recorded in 16.7% patients in OG and 45% in LG (*p* = 0.03). The remaining demographic data, disease behaviour, and comparison between groups are summarised in (Table 1). Forty-two (67.7%) patients received biologics preoperatively. The statistically significant difference in the higher use of immunomodulators (IS) in the OG is very likely due to the more frequent use of infliximab as a first-line biologic, which is standardly given in combination with IS, as opposed to adalimumab, which is more often given as monotherapy [13]. The administration of concomitant medication in these groups is summarised in (Table 2). No patient underwent bowel preparation. All patients were given broad-spectrum antibiotic prophylaxis and continued postoperatively. Median duration of the surgery was 130 min (IQR 115–154) in OG and 148 min (IQR 126–170) in LG (*p* = 0.07). Fifteen patients in LG underwent ICR surgery via SILS. All ileocecal anastomoses in OG were fashioned as end-to-end in LG were used two anastomosis types—end-to-end 14 (70%) and, from September 2020, side-to-side. Conversion to open surgery was reported once, in a patient who underwent ICR and resection of enterocolic fistula. In the postoperative period, the patient's oral intake was started significantly earlier in patients in LG than in OG (*p* < 0.0001). The remaining data on postoperative monitored parameters as well as a comparison between the groups are summarised in (Table 3). There were three (7.14%) postoperative complications in OG and one (5%) in LG according to CDc. Reported complications in OG were one intra-abdominal abscess, one enterocutaneous fistula and one wound infection. An intra-abdominal abscess was recorded as the complication in LG in patients who underwent semi-selective ICR after CT-guided drainage of an intra-abdominal abscess. There was no significant difference in the number of complications depending on the type of the operation (*p* = 1). The postoperative complication rate did not differ between types of surgery even when adjusted for other predictors even after adjusting for the type of laparoscopic approach or the type of intestinal anastomosis (Table 4). There was no ROR and no case of ileus/bowel obstruction in both groups. None of the patients experienced non-SSIs (urinary tract infection, pneumonia, or bacteremia). Median length of hospitalisation was significantly shorter in LG (LG 7, IQR 6–8 days; OG 8, IQR 7–10.3 days; *p* = 0.0005). Median length of follow-up was 21.5 months (IQR 12.8–40.3). The absolute 30-day mortality rate was zero. All postoperative data as well as a comparison between the groups are summarised in (Table 5).

**Table 1:** Demographic variables for the entire cohort

Variable	Overall, *n* = 62	OG, *n* = 42 (67.7%)	LG, *n* = 20 (32.3%)	*p* value
	*n* (%)	*n* (%)	*n* (%)	
Female	30 (48.4)	19 (45.2)	11 (55)	0.58
Age at surgery, years (median, IQR)	16.67 (14.75–17.41)	16.78 (15.59–17.41)	16.19 (13.92–17.38)	0.23
Age at diagnosis, years (median, IQR)	13.74 (10.96–15.98)	14.05 (11.58–16.01)	13.12 (8.88–15.86)	0.46
Disease duration, months (median, IQR)	23 (6.50–44.50)	28 (7.50–43.0)	14 (3.25–59.25)	0.04
Diabetes mellitus	0 (0)	0 (0)	0 (0)	> 0.99
BMI, kg/m^2^ (median, IQR)	19.37 (16.74–21.88)	19.38 (17.22–22.28)	18.81 (15.36–20.69)	0.29
BMI				
< 18	24 (38.71)	15 (35.71)	9 (45.0)	0.58
18–24	29 (46.77)	22 (52.38)	7 (35.0)	0.28
25–29	3 (4.84)	2 (4.76)	1 (5)	> 0.99
30–34	0 (0)	0 (0)	0 (0)	> 0.99
> = 35	1 (1.61)	1 (2.38)	0 (0)	> 0.99
Disease behaviour				
B1 (non-stricturing/penetrating)	12 (19.35)	9 (21.43)	3 (15.0)	0.74
B2 (stricturing)	34 (54.84)	26 (61.90)	8 (40.0)	0.17
B3 (penetrating)	16 (25.81)	7 (16.67)	9 (45.0)	0.03
Perianal modifier	11 (17.74)	8 (19.05)	3 (15.0)	> 0.99

Data are presented as numbers (%) unless otherwise noted

*BMI* body mass index; *IQR* interquartile range; *LG* laparoscopic group; *OG* open group

**Table 2:** Preoperative medications for the entire cohort

Variable	Overall, *n* = 62	OG, *n* = 42 (67.7%)	LG, *n* = 20 (32.3%)	*p* value
	*n* (%)	*n* (%)	*n* (%)	
Corticosteroids	1 (1.61)	1 (2.38)	0 (0)	> 0.99
Immunomodulators	49 (79.03)	37 (88.10)	12 (60)	0.02
Biologics	42 (67.74)	26 (61.90)	16 (80.0)	0.25

Data are presented as numbers (%) unless otherwise noted

*LG* laparoscopic group; *OG* open group

**Table 3:** Postoperative monitored parameters for the entire cohort

Variable	Overall, *n* = 62	OG, *n* = 42 (67.7%)	LG, *n* = 20 (32.3%)	*p* value
	Median (IQR)	Median (IQR)	Median (IQR)	
Postoperative length of stay, day	8 (7–10)	8 (7.0–10.25)	7 (6–8)	0.0005
Epidural catheter—POD	3 (3.0–4.0)	4.0 (3.0–4.0)	3.0 (2.0–3.0)	0.009
VAS 0. POD	3 (0–4.0)	3.0 (0–4.0)	3.0 (0–3.0)	0.37
VAS 1. POD	1.0 (0–3.5)	2 (0–4.0)	0 (0–3.0)	0.08
VAS 2. POD	0 (0–1.0)	0 (0–1.0)	0 (0–1.75)	0.89
VAS 3. POD	0 (0–0)	0 (0–1.0)	0 (0–0)	0.17
VAS overall	0 (0–3.0)	0 (0–3.0)	0 (0–3.0)	0.08
Fluids intake—POD	2.0 (1.0–3.0)	3.0 (1.0–3.0)	1.0 (1.0–2.0)	< 0.0001
Soft food intake—POD	4.0 (3.0–4.0)	4.0 (4.0–5.0)	2.0 (2.0–3.75)	< 0.0001
UC—POD	4.0 (3.0–4.0)	4.0 (3.0–5.0)	3.0 (3.0–4.0)	0.0003
CVC—POD	7.0 (6.0–8.0)	7.0 (6.5–8.0)	5.0 (4.0–6.0)	< 0.0001
PN—PO	5.0 (4.0–6.0)	5.0 (5.0–6.0)	4.0 (3.0–4.0)	< 0.0001
ATB—POD	7.0 (6.0–8.0)	7.0 (7.0–8.0)	6.0 (5.0–6.75)	< 0.0001

Data are presented as numbers (median, IQR) unless otherwise noted

*CVC* central venous catheter; *IQR* interquartile range; *LG* laparoscopic group; *OG* open group; *PN* parenteral nutrition; *POD* postoperative day; *UC* urinary cathetrisation; *VAS* visual analogue scale

**Table 4:** Multivariate analysis of associations between type of surgery and postoperative complications

Variable	OR	95% CI	*p* value
Surgical approach	1.79	0.16–44.56	0.65
Disease behaviour	3.59	0.33–39.71	0.28
Immunomodulators	Inf	0.00–NA	0.23
Disease duration	0.99	0.93–1.04	0.80

Disease behaviour is according to the Paris classification

*CI* confidence interval; *OR* odds ratio

**Table 5:** Postoperative complications and follow-up variables for the entire cohort

Variable	Overall, *n* = 62	OG, *n* = 42 (67.7%)	LG, *n* = 20 (32.3%)	*p* value
	*n* (%)	*n* (%)	*n* (%)	
Postoperative length of stay, day (median, IQR)	8 (7–10)	8 (7.0–10.25)	7 (6–8)	0.0005
Mortality	0 (0)	0 (0)	0 (0)	> 0.99
SSI (S, D, OS)	4 (6.45)	3 (7.14)	1 (5.0)	> 0.99
non-SSI (UTI, pneumonia, bacteremia)	0 (0)	0 (0)	0 (0)	> 0.99
CDc: minor complication	1 (1.61)	1 (2.38)	0 (0)	> 0.99
CDc: major complication	3 (4.84)	2 (4.76)	1 (5.0)	> 0.99
IASC	3 (4.84)	2 (4.76)	1 (5.0)	> 0.99
Ileus	0 (0)	0 (0)	0 (0)	> 0.99
Bleeding	0 (0)	0 (0)	0 (0)	> 0.99
Readmission	0 (0)	0 (0)	0 (0)	> 0.99
Reoperation	0 (0)	0 (0)	0 (0)	> 0.99
Follow-up, months (median, IQR)	21.5 (12.25–40.75)	26.0 (15.25–44.75)	16.0 (6.0–23.0)	0.04

Data are presented as numbers (%) unless otherwise noted

*CDc* the Clavien–Dindo classification (minor–I or II); *D* deep; *IASC* intra-abdominal septic complication; *Ileus* bowel obstruction with radiographic confirmation and requirement of a nasogastric tube for decompression or operative intervention; *IQR* interquartile range; *LG* laparoscopic group; *non-SSI* nonsurgical-site infections; *OG* open group; *OS* organ/space; *ROR* return to the operating room; *S* superficial; *SSI* surgical-site infection; *UTI* urinary tract infection

## Discussion

To the best of our knowledge, this study is performed on the largest cohort of consecutive paediatric CD patients, who underwent ICR and presents data from recent laparoscopic and medical treatment period. The latest study on this topic is from 2020, but it follows the period 2009–2012 [14]. The exceptional value of our results lies in the fact that they were obtained at a time when the latest published recommendations for the surgical treatment of CD in children (2017) refer to laparoscopy, but cannot give a clear statement due to lack of data.

Our analysis shows that the laparoscopic-assisted approach did not increase the risk of infectious and non-infectious postoperative complications according to CDc, readmission rate or reoperation. Moreover, laparoscopic group experienced a reduction in the length of hospitalisation, realimentation, urinary catheterisation and use of epidural analgesia.

### Minimally invasive surgery (MIS)

#### Standard laparoscopy

MIS is the method of choice for a large number of surgical procedures in adult patients, and its use is gradually expanding in paediatric surgery as well. The slower expansion of MIS in paediatric patients is due to several factors—anatomical proportions of the patient’s smaller body, smaller size of paediatric populations and lack of laparoscopic training [15]. The risk factor that is specifically associated with CD is intra-abdominal inflammation (thickened mesentery, phlegmons, fistulas, adhesions and abscesses) which can make a minimally invasive approach challenging. Despite all these pitfalls, laparoscopy has advantages for which it is recommended as the first-line approach in adult surgery for Crohn’s disease [6]. Our analysis shows a trend towards more frequent use of MIS also in paediatric surgery. Since 2018, all patients have undergone laparoscopic-assisted ICR, no statistically significant prolongation of the operation time was recorded, and despite the fact that in LG 45% of patients had a complex CD, the conversion rate was minimal.

#### Single-incision laparoscopic surgery

Following on from the above with growing experience with laparoscopy [16], the single incision laparoscopic surgery (SILS) approach is becoming a common part of laparoscopy for CD in adult patients [17–19]. Moreover, the SILS has been introduced in paediatric surgery for CD with promising results [20]. At the author’s institution, the SILS programme for CD began in May 2019 and since then, all patients consecutively underwent SILS ICR. Until the final data collection, 15 operations were performed using the SILS approach. One-third of patients had complex CD. Preliminary results are promising and comparable with literary data.

### Complications

IBD surgery is burdened by a greater number of complications than comparable operations for other conditions, with the fact that not only the surgical approach, but also preoperative medical treatment, including recently available biological treatment, can contribute to their development [10, 11]. According to the literature, early postoperative complications occur in up to 30% of cases during surgery on the small intestine or in the ileocecal region [21].

#### Complications and laparoscopy

The most recent work on this topic is an US study from 2020, which, according to The Kids’ Inpatient Database, evaluated and compared complications in 760 paediatric patients after an open and laparoscopic ICR using propensity score matched analysis for the time period 2009–2012. When comparing wound infection, there is no statistically significant difference depending on the approach to ICR, unfortunately, due to the nature of the study, important perioperative factors, such as operative time and current medical therapies, are not included in the comparison [14].

Diederen et al. retrospectively assessed complications after ICR from 7 tertiary paediatric centres in the Netherlands between 1990 and 2015. The study consists of 122 patients (B3 form 11.5%), 65% of them underwent laparoscopic ICR—0.7 ICR per year at one surgical department. The overall complication rate was 29.5% and according to the analysis, the operative approach had no effect on the severe (*p* = 0.146) or intra-abdominal septic complications (*p* = 0.114) [22].

Overall, the number of complication within our dataset is comparable or even lower than reported in the literature [14, 22–29] even considering the fact that in our group, more than a quarter of patients (25.8%) had a complex form of CD, which was recorded even in 45% of patients in the LG.

One of the weaknesses of all paediatric surgery studies on postoperative complications is that the overall number of patients treated is small. However, meta-analyses can give stronger conclusions but only if the complication values are comparable and therefore a uniform classification such as the Clavien—Dindo classification [12] is necessary to use. This classification is a widely accepted tool used to assess and report all postoperative complications, but mainly in general surgery. Only one of the above-mentioned studies following MIS in the paediatric CD population used this classification [22]. Rather, they applied several heterogeneous systems.

#### Complications and biologics

In our study, preoperative biologic therapy with anti-TNF alpha did not affect the incidence of postoperative complications. This is not consistent with the latest recommendation for the treatment of CD in the paediatric population, according to which biologics should be discontinued before surgery [7], but it is in agreement with recent studies on this topic [6, 30, 31]. Direct measurement of drug levels in intestinal tissues could help to further understand the effect of biological therapy on possible postoperative complications.

### Hospitalisations and other postoperative results

There is considerable debate in the literature about use of MIS for CD for many different reasons, but as with many other conditions, when laparoscopic techniques have been used in Crohn’s disease surgery in adults and children, they have been shown to result in shorter hospital stays, faster return to a regular diet, less postoperative narcotic use, and better cosmetic results [14, 23, 32]. These results have been confirmed also in our analysis—postoperative oral intake was started significantly earlier in patients in LG than in OG (LG 1 days versus OG 3 days, *p* < 0.0001) as well as central venous catheter (*p* < 0.0001), parenteral nutrition (*p* < 0.0001), postoperative urinary cathetrisation (*p* < 0.0001) and need of epidural analgesia (*p* = 0.009) were shorter in LG, the length of hospitalisation was also significantly shorter in LG (*p* = 0.0005).

## Limitations

The main limitation of our study is its retrospective design and the fact that it is single centre study form the tertiary centre. Partial heterogeneity in the LG, which arose on the basis of following current trends and recommendations in IBD surgery, was verified by multivariate analysis.

Despite these limitations, this is a study where the operation was performed by a single operating team, and all consecutive patients who underwent ICR as a restorative procedure were included, laparoscopy was already a common part of the surgical approach in children, the evaluation period thus corresponds to the most current recommendations of medical and surgical treatment and due to the nature of the workplace and the team of authors, it involves direct monitoring of patients with the possibility of tracing almost all their data.

## Conclusions

Our data confirmed the hypothesis that the laparoscopic approach in paediatric patients with Crohn’s disease has no effect on postoperative complications, the risk of reoperation or readmission. In addition, in the LG, there was a significant reduction in the length of hospitalisation and the placement of urinary catheter, central venous and epidural catheter. Therefore, laparoscopic surgery should be considered the preferred surgical approach for primary ileocecal resection in children with Crohn’s disease.

## Data Availability

The data are available.
